# Feasibility of Using an Electrolysis Cell for Quantification of the Electrolytic Products of Water from Gravimetric Measurement

**DOI:** 10.1155/2018/2681796

**Published:** 2018-02-05

**Authors:** Samuel Melaku, Zewdu Gebeyehu, Rajeev B. Dabke

**Affiliations:** Department of Chemistry, Columbus State University, Columbus, GA 31907, USA

## Abstract

A gravimetric method for the quantitative assessment of the products of electrolysis of water is presented. In this approach, the electrolysis cell was directly powered by 9 V batteries. Prior to electrolysis, a known amount of potassium hydrogen phthalate (KHP) was added to the cathode compartment, and an excess amount of KHCO_3_ was added to the anode compartment electrolyte. During electrolysis, cathode and anode compartments produced OH^−^(aq) and H^+^(aq) ions, respectively. Electrolytically produced OH^−^(aq) neutralized the KHP, and the completion of this neutralization was detected by a visual indicator color change. Electrolytically produced H^+^(aq) reacted with HCO_3_^−^(aq) liberating CO_2_(g) from the anode compartment. Concurrent liberation of H_2_(g) and O_2_(g) at the cathode and anode, respectively, resulted in a decrease in the mass of the cell. Mass of the electrolysis cell was monitored. Liberation of CO_2_(g) resulted in a pronounced effect of a decrease in mass. Experimentally determined decrease in mass (53.7 g/Faraday) agreed with that predicted from Faraday's laws of electrolysis (53.0 g/Faraday). The efficacy of the cell was tested to quantify the acid content in household vinegar samples. Accurate results were obtained for vinegar analysis with a precision better than 5% in most cases. The cell offers the advantages of coulometric method and additionally simplifies the circuitry by eliminating the use of a constant current power source or a coulometer.

## 1. Introduction

The quantity of a substance produced at the electrode and the quantity of electric charge passed are linked with Faraday's laws of electrolysis. Many reports present coulometric methods of analysis of a variety of reagents [1–17]. A number of original reports [3, 4, 11, 15–17] and reviews [1, 2, 5–10, 12–14] on coulometric methods were published over the past decades. Coulometric method has been applied to analyze a large variety of inorganic and organic compounds [1, 6, 13]. Standardization of reagents is not required in coulometric analysis as the quantity of reagent prepared is directly determined from the charge passing through the cell.

Alternatively, the quantification of a reagent produced in an electrolysis cell without direct monitoring of charge is also well known [5]. This work includes early research on estimating the charge passing through the cell from measuring the mass of silver deposited on the platinum electrode [18, 19]. Similarly, the measurement of color change has been applied to quantify the charge passing through the cell [20]. This method was employed for monitoring the reduction of permanganate ions at platinum electrode and the oxidation of copper to blue cupric triethanolamine complex. Although these methods sidestep the use of a coulometer or a constant current source, they had practical constraints for routine analytical purposes due to the involvement of large-size precious metal electrodes or a rotating electrode placed in a special type of electrochemical cell.

Simple measurement of the volumes of hydrogen and oxygen gases produced at the cathode and anode in an electrolysis cell offers advantages of coulometric method without actually using a coulometer or any electronic equipment. In fact, early studies indicated that hydrogen-oxygen coulometer was easy to assemble and capable of measuring the quantity of electric charge with an accuracy of ±0.1% or better [4]. By choosing a proper size buret for monitoring the volume of gases, the instrument was readily adapted to measuring diverse quantities of electricity down to about 10 coulombs [5]. Enhanced current efficiency was noted for the hydrogen–nitrogen coulometer [21] in which hydrazine sulfate was used as an electrolyte responsible for producing nitrogen gas instead of oxygen gas at the platinum anode. An electrolysis cell was used to measure the volume of O_2_(g) and monitor an acid-base titration [22].

As stated in (1), electrolysis of water produces OH^−^(aq) in the cathode compartment. In a 1 : 1 stoichiometric ratio (2), electrolytically produced OH^−^(aq) neutralizes the weak acid (e.g., potassium hydrogen phthalate (KC_8_H_4_O_4_H) or C_8_H_4_O_4_H^−^ in an anionic form) added to this compartment:

(1) $$\begin{array}{c} 2{\text{H}}_{2}\text{O}\left(\text{l}\right)+2e\to 2{\text{OH}}^{-}\left(\text{aq}\right)+{\text{H}}_{2}\left(\text{g}\right) \end{array}$$

(2) $$\begin{array}{c} {\text{C}}_{8}{\text{H}}_{4}{\text{O}}_{4}{\text{H}}^{-}\left(\text{aq}\right){+\text{OH}}^{-}\left(\text{aq}\right)\to {\text{C}}_{8}{\text{H}}_{4}{\text{O}}_{4}^{2-}\left(\text{aq}\right){+\text{H}}_{2}\text{O}\left(\text{l}\right) \end{array}$$

Concurrently, anode produces H^+^(aq) the following equation:

(3) $$\begin{array}{c} {\text{H}}_{2}\text{O}\left(\text{l}\right)\to {2\text{H}}^{+}\left(\text{aq}\right)+\frac{1}{2}{\text{O}}_{2}\left(\text{g}\right)+2\text{e} \end{array}$$

Electrolysis of water in cathode and anode compartments (1) and (3) also produces H_2_(g) and O_2_(g) at the respective electrodes. A chemical reaction (4) between electrolytically produced H^+^(aq) and externally added KHCO_3_(aq) produces CO_2_(g):

(4) $$\begin{array}{c} {\text{H}}^{+}\left(\text{aq}\right){+\text{HCO}}_{3}^{-}\left(\text{aq}\right)\to {\text{H}}_{2}\text{O}\left(\text{l}\right){+\text{CO}}_{2}\left(\text{g}\right) \end{array}$$

When a known quantity of KHP (a primary standard substance) is added to the cathode compartment, the endpoint of the neutralization reaction between electrolytically produced OH^−^(aq) and KHP can be determined visually with use of phenolphthalein as an indicator. Electrolysis is promptly stopped at the endpoint. Prior to electrolysis, an excess amount of KHCO_3_ is added to the anode compartment.

Concurrent liberation of chemically produced CO_2_(g) in addition to H_2_(g) and O_2_(g) amplified the effect of decreasing in the mass. Mass of the cell is continuously monitored before, during, and after electrolysis. From the mass change experienced by the cell, the amount of KHP can be determined to assess the stoichiometric relations between the electrolytic products of water. In this study, the feasibility of monitoring the mass change experienced by an electrolysis cell due to the liberation of gases and quantifying the electrolytic products of water was assessed.

## 2. Materials and Methods

### 2.1. Chemicals and Materials

Reagent-grade potassium nitrate, KHP, potassium hydrogen carbonate, and agar were obtained from Fisher. Millipore deionized water was used for preparation of reagents. Alfa Aesar platinum wire (0.762 mm diameter) was used as purchased. 0.1% phenolphthalein was used as a visual indicator to detect the completion of neutralization. Ohaus Pioneer balance (Model: PA114, 0.0001 g readability, and ±0.0002 g linearity) was used for mass measurements. Vinegar samples were locally purchased from a grocery store.

### 2.2. Electrolysis Cell

**Figure 1:**
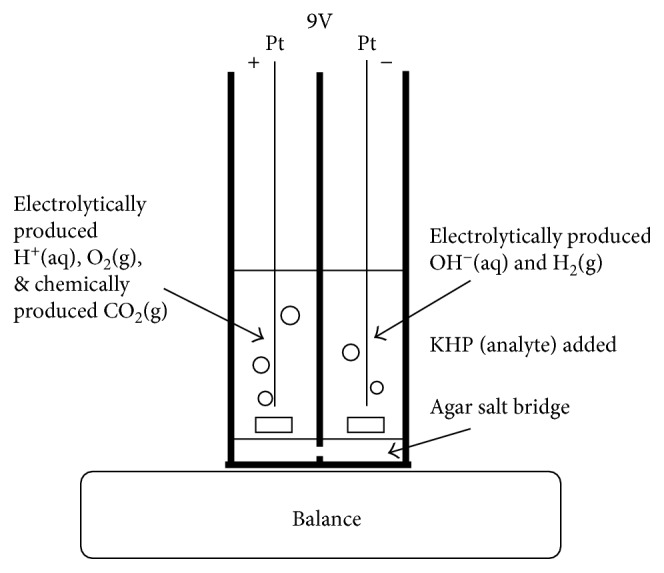
Schematic diagram of the water electrolysis cell. KNO_3_(aq) was used as an electrolyte in both compartments. KHCO_3_(aq) was added to the anode compartment. Effective concentrations of KNO_3_ and KHCO_3_ were 1 M each. Desired number of moles of KHP(aq) and a drop of 0.1% phenolphthalein were added to the cathode compartment. The volumes of electrolyte in each compartment were approximately 3.2 mL. Platinum wires were immersed in the electrolytes and directly connected to two 9 V batteries connected in parallel. The cell was placed on a digital balance housed in a glass cabinet.

Electrolysis cell was made of spectrophotometer plastic cuvettes. A 3 mm hole was drilled on a side near the base of each cuvette, and the cuvettes were glued together using an epoxy. Bottom portions of two additional cuvettes were cut and glued on top (Figure 1). The extra depth offered by these cuvettes prevented the mass loss due to the spattering of electrolyte mist formed due to liberation of gas bubbles during electrolysis. A mixture of agar and potassium nitrate (3% by mass and 1 M, resp.) was added to water, and the mixture was heated to 90°C. A few drops of this mixture were placed on the bottom of the cuvettes. A gel was formed on the bottom of the cuvettes, serving as a salt bridge. KNO_3_(aq) was used as an electrolyte in both compartments. KHCO_3_ was added to the anode compartment. Effective concentrations of KNO_3_ and KHCO_3_ were 1 M each. Platinum wires served as cathode and anode and were directly powered by two 9−V batteries connected in parallel. Approximately 3.5 cm platinum wire was dipped in the electrolyte in each compartment. The current passing through the cell was in the range of 55 mA to 90 mA. A multimeter was used to display the current passing through the electrolysis cell. Two miniature magnetic stir bars were placed in the compartments, and an external stir bar retriever was used to maneuver the internal stir bars in up and down directions. The movement of stir bars helped dislodge the gas bubbles adhering to the electrodes and sidewalls and stir the electrolytes in both compartments. Additionally, electrolyte stirring assisted uniform mixing of the contents of cathode compartment. This action was periodically performed, particularly before mass measurement.

### 2.3. Determination of Mass Correction Factor

**Figure 2:**
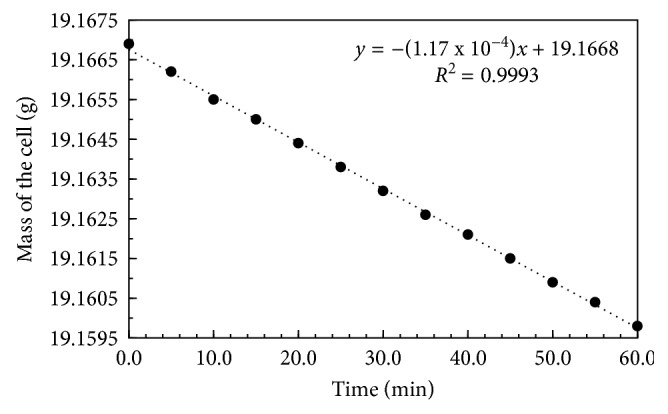
Monitoring the mass of an idle cell (no electrolysis).

Mass correction factor was determined to quantify an error associated with the mass loss due to the evaporation of electrolytes. In this experiment, mass of an idle cell (no electrolysis) was monitored at an interval of 5 minutes. Mass of the cell was monitored for 60 minutes (Figure 2).

### 2.4. Electrolytic Titrations of KHP

**Figure 3:**
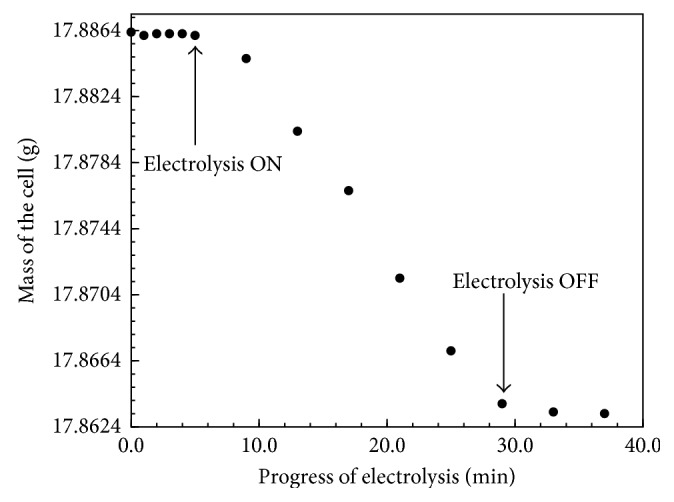
Monitoring the mass of the electrolysis cell before, during, and after electrolysis.

**Figure 4:**
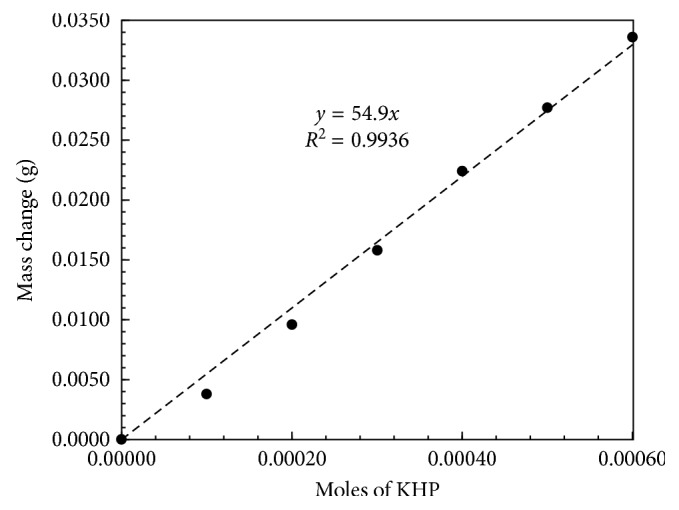
A plot of the mass change experienced by the cell versus number of moles of KHP neutralized in the cathode compartment.

Desired moles of KHP were added to the cathode compartment. Mass of the cell was monitored at a time interval of one minute for the first five minutes before electrolysis. After this initial rest period, electrolysis was started. Electrolysis was paused at a two-minute time interval, stir bars were maneuvered in up and down position, and the mass was recorded after a two-minute rest period. Electrolysis was promptly stopped when the cathode compartment electrolyte turned light pink indicating an endpoint of the titration. Mass measurement was continued for additional eight minutes. The results of this experiment are presented in Figure 3. Electrolytic titration of KHP was repeated to determine the mass change experienced by the cell for various concentrations (0 to 6.0 × 10^−4^) of KHP (Figure 4).

### 2.5. Coulometric Measurements

**Figure 5:**
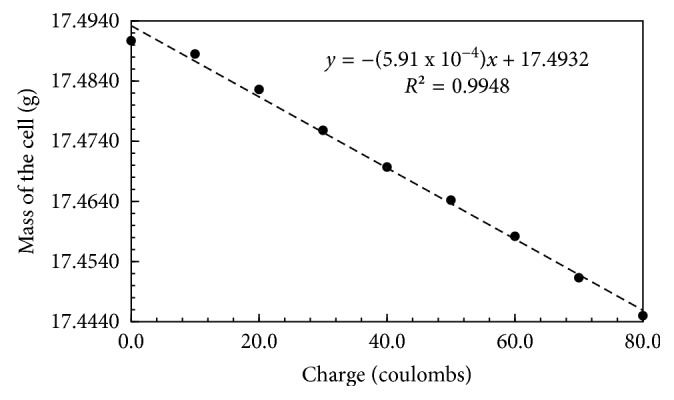
A plot of the mass of the electrolysis cell versus the charge passing through the cell.

In view of confirming the stoichiometric relations between the moles of OH^−^(aq) and the mass loss due to the liberation of electrolytically produced H_2_(g) and O_2_(g) and chemically produced CO_2_(g), we monitored the charge passing through the electrolysis cell on Faraday MP potentiostat. Electrolysis was paused at 10 coulombs interval, stir bars were maneuvered in up and down position, and the mass was recorded after one-minute rest period (Figure 5).

### 2.6. Electrolytic Titrations of Real Sample

A known volume of commercial vinegar sample was added to the cathode compartment and titrated against electrolytically produced OH^−^(aq). The quantity of acid in a sample was determined from the mass change experienced by the cell.

## 3. Results and Discussion

### 3.1. Mass Correction Factor

The mass of an idle cell linearly decreased with time (Figure 2). A linear decrease in mass indicated uniform mass loss resulting from the evaporation of electrolyte. The slope of this plot quantified the mass correction factor of 1.17 × 10^−4^ g/min or 1.95 × 10^−6^ g/s. This factor was used to determine the mass loss due to evaporation during the period for each trial of the experiment.

### 3.2. Quantitative Assessment of the Electrolytic Products

Faraday's laws of electrolysis and (1) and (3) indicate, on passing of one Faraday charge through water, the electrolysis cell produces one mole of OH^−^(aq) and H^+^(aq) in their respective compartments. Additionally, 0.5 moles of H_2_(g), 0.25 moles of O_2_(g), and 1 mole of CO_2_(g) are liberated. Considering molar masses of these gases, a decrease in mass by 53.0 g was expected to result from passing one Faraday of charge.

Figure 3 presents data on the mass of the cell before, during, and after electrolytic titration. Desired moles (4.0 × 10^−4^ moles) of aqueous KHP were added to the cathode compartment, and the charge was passed through the cell. Mass of the cell significantly decreased during the course of electrolysis. Mass of the cell slightly decreased prior to electrolysis, resulting from the evaporation of electrolyte, and it continued to decrease after electrolysis. Mass correction factor was applied to minimize the error associated with the evaporative mass losses during the course of electrolysis.

The electrolytic titration experiment presented in Figure 3 was repeated for various concentrations of KHP, and the mass correction factor was independently applied to each trial of experiment. A linear response of mass change versus moles of KHP confirmed the quantitative relationship between the moles of KHP and the drop in the mass due to the liberation of gases (Figure 4). Slope of the plot indicated a mass drop of 54.9 g due to the liberation of gases. This mass accounted for the electrolytic neutralization of one mole of KHP after passing one Faraday charge. This quantity of mass obtained from the slope matched with the estimated mass loss of 53.0 g/Faraday. A blank trial (a data point corresponding to zero moles of KHP in Figure 4) confirmed minimal contribution of acidic impurities and dissolved CO_2_(g) present in the electrolyte. Literature values [23] of solubility of H_2_(g), O_2_(g), and CO_2_(g) at 1 atm pressure and at 25°C temperature also confirm a minor error in mass measurement (<0.15%) associated with the dissolution of gases produced in the cell. The trials presented in Figure 4 were repeated to determine the relative standard deviation in the slope. The RSD value (0.93%, *n* *=* 3) signified a reliable quantitative relationship between the mass change and the moles of KHP.

A linear response of the plot (Figure 5) confirmed the mole stoichiometry presented in (1), (3), and (4). A slope of this plot indicated that 5.91 × 10^−4^ g mass drop occurred per coulomb charge passed through the cell. Accounting for the mass loss due to evaporation of the electrolyte, a correction factor (1.95 × 10^−6^ g/s) was applied to the slope. Considering an estimated time (17.5 s) required to pass one coulomb charge, mass loss due to evaporation during the passage of one coulomb was 3.41 × 10^−5^ g/C. The mass-corrected slope after deducting this factor was 5.57 × 10^−4^ g/C or 53.7 g/Faraday. This mass drop was in agreement with the estimated mass drop of 53.0 g/Faraday determined from the stoichiometric relation presented in (1–4). A linear relation between the mass loss due to liberation of gases and the charge passed through the cell (Figure 5) indicated negligible interference of dissolution of gases produced during electrolysis. The slope of the plot presented in Figure 5 was confirmed from three independent experiments (RDS 0.49%).

### 3.3. Analysis of Real Sample

The efficacy of the electrolytic titration method presented in this paper was tested for the analysis of acid content in household vinegar samples. A known volume (500 μL) of vinegar was added to the cathode compartment and titrated against electrolytically produced OH^−^(aq) as presented in the experimental section. The molarity and percent acid content in the sample were determined from the mass change experienced by the cell (Table 1). The mass correction factor was independently applied to each trial. Quantity of acid determined from electrolytic titration was in agreement with the manufacturer's label, and the quantities were consistent with three household vinegar samples.

## 4. Conclusions

In this work, the feasibility of using an electrolysis cell for quantification of the electrolytic products of water from gravimetric measurement was tested. The cell presented in this paper enables in situ production of reagents and their direct quantification and does not require standardization of reagents. The electrolysis cell directly powered by 9 V batteries eliminates the requirement of a constant current source or a coulometer, yet offers advantages of coulometric method of titration. The cell utilizes minimal volume of reagents (3.2 mL or less in each compartment). The electrolysis cell is simple, transparent, and easy to fabricate. The cell eliminates the requirement of an external salt bridge or a fritted glass membrane. Linear response of the decrease in the mass of the cell to the moles of KHP added and to the charge passing through the cell validates the applicability of the method. An agreement between the estimated drop in mass (determined from Faraday's laws of electrolysis) and the experimentally determined drop in mass underlines the feasibility of using the electrolysis cell for quantification of the electrolytic products. With acceptable values of relative standard deviation (better than 5% in most cases), the mass drop experienced by the cell quantitatively responds to the acid content in household vinegar samples.

## Figures and Tables

**Table 1:** Results of titration of an acid in commercial vinegar samples by electrolytically produced OH^−^(aq).

Percent acid content stated on manufacturer's label	Mass change in electrolysis cell (g)	Moles of acid in (mmol)	Average molarity	Percent acid content determined from this study (95% CI, *n* *=* 3)	Percent RSD
4	0.0165	0.311	0.623	3.74% (±1.32)	14.3
5	0.0222	0.419	0.838	5.03% (±0.53)	4.3
7	0.0305	0.575	1.15	6.91% (±0.52)	3.0
